# Influence of Pulse Energy and Defocus Amount on the Mechanism and Surface Characteristics of Femtosecond Laser Polishing of SiC Ceramics

**DOI:** 10.3390/mi13071118

**Published:** 2022-07-15

**Authors:** Xuanhua Zhang, Xiaoxiao Chen, Tao Chen, Guiying Ma, Wenwu Zhang, Lirong Huang

**Affiliations:** 1 School of Mechanical and Electrical Engineering, Jiangxi University of Science and Technology, Ganzhou 341000, China; zhangxuanhua@nimte.ac.cn (X.Z.); chentao11@nimte.ac.cn (T.C.); 2 Ningbo Institute of Materials Technology and Engineering, Chinese Academy of Sciences, Zhejiang Provincial Key Laboratory of Aero Engine Extreme Manufacturing Technology, Ningbo 315201, China; maguiying@nimte.ac.cn (G.M.); zhangwenwu@nimte.ac.cn (W.Z.); 3 University of Chinese Academy of Sciences, Beijing 100049, China; 4 School of Mechanical Engineering and Mechanics, Ningbo University, Ningbo 315211, China

**Keywords:** femtosecond laser polishing, SiC ceramics, laser ablation, surface quality

## Abstract

SiC ceramics have excellent comprehensive properties and are typical hard and brittle materials that are difficult to process and are widely used in many fields. Laser polishing technology has developed into a new surface processing technology, and femtosecond laser polishing has become an important method for the precision machining of hard and brittle materials. In this paper, SiC ceramics were ablated and polished by infrared femtosecond laser, the laser ablation threshold of SiC ceramics was calculated and the influence of pulse energy and defocus amount on the surface morphology, surface roughness, polishing depth and oxidation degree of femtosecond laser polishing of SiC ceramics were investigated. The results show that when the laser repetition frequency *f* = 175 kHz, wavelength *λ* = 1064 nm and ablation time *t* = 9 s, the laser ablation threshold of SiC ceramics is 0.355 J/cm^2^. With the increase in pulse energy, the surface roughness first decreased and then increased, and the polishing depth showed an overall upward trend. The change of defocus amount will lead to the change of the laser spot diameter. With the increase of the defocus amount, the laser spot irradiated on the workpiece surface becomes larger, and the laser energy density decreases, which results in the decrease of the laser ablation ability and polishing depth and the increase of the polished surface roughness. Periodic nano-ripple structures appeared on the laser-induced surface. Through Energy Dispersive Spectrometer (EDS) elemental analysis, it was found that there was an oxidation phenomenon in SiC ceramics polished by femtosecond laser in an air environment, and the change of pulse energy and defocus amount had insignificant effects on the degree of oxidation.

## 1. Introduction

SiC ceramics can adapt to extreme working environments due to their excellent thermal shock resistance, high strength at high temperature, low thermal expansion coefficient, excellent corrosion resistance and low density, which have huge application prospects in the fields of aerospace, space optics, semiconductors and high-temperature components [1,2,3]. With the gradual development of SiC ceramic–related products in the direction of high-precision and high-quality, higher requirements for the surface quality of SiC ceramics have been put forward. Due to the poor surface quality of SiC ceramics prepared by sintering technology and SiC ceramics being hard and brittle materials with high hardness and brittleness, it is very difficult to obtain high-precision SiC ceramic surfaces by traditional processing methods, which limits the application of SiC ceramics in the precision manufacturing field.

The traditional surface precision machining technologies of SiC ceramics include mechanical polishing [4], ELID grinding [5], plasma polishing [6], chemical mechanical polishing [7], magnetorheological polishing [8], etc. These processing methods have defects such as low processing quality, low processing efficiency, high cost and environmental pollution, which make it difficult to meet actual needs [9]. Laser polishing technology is non-contact polishing, which can not only effectively avoid the above defects but also has the advantages of high flexibility, easy combination with CNC technology to realize automation, a wide processing range and suitability for the surface polishing of complex parts such as planar, spherical and free-form surfaces, making it a surface precision machining method for hard and brittle materials with application prospects and development potential [10]. Considering the complex interaction mechanism between laser and materials involving complex physical and chemical processes, the laser polishing mechanism can be divided into thermal effect and photochemical decomposition (such as thermal polishing and cold polishing) [11]. Thermal polishing generally uses continuous wave lasers or medium- and long-pulse lasers. When the laser beam is radiated to the surface of material, the material absorbs laser energy, causing the local area temperature to rise rapidly. When the energy density of the laser spot reaches a certain level, the material in the irradiated area melts or evaporates, and the surface roughness is reduced by material remelting distribution or removal of the material. Domestic and foreign scientific researchers have conducted more research on laser thermal polishing, which is more suitable for polishing metal materials. Miller et al. [12] used the continuous wave laser to polish H13 die steel and found when the optimal transient combination of laser power and scanning speed was used, surface quality improvement of 83% was obtained. Ma et al. [13] used the fiber laser to polish additive-manufactured Ti-based alloy surfaces. The results showed that the surface roughness, wear resistance and microhardness after laser polishing were better than those of the original surface. Xu et al. [14] used a continuous wave laser and a nanosecond pulsed laser to polish the surface of TiAl alloy fabricated by laser deposition and studied the differences in surface morphology, microstructure, microhardness, corrosion resistance and wear resistance between the two polishing processes. Gao et al. [15] used a nanosecond laser to ablate SiC ceramics and found that when the laser energy is low, the material is removed by evaporation. When the incident laser energy is high, the material removal mechanism is liquid phase explosion, producing a splash of liquid around the ablation area. Some studies have shown that when a continuous wave laser or a medium- and long-pulse laser was used to polish hard and brittle materials, due to the large heat-affected zone, defects such as debris deposition, microcracks and oxidation were prone to occur [16]. Therefore, it is difficult for laser thermal polishing to meet the surface precision machining requirements of hard and brittle materials.

Compared with continuous wave lasers and medium- and long-pulse lasers, ultrashort-pulse lasers have the advantages of small heat-affected zone, fewer thermal defects and high machining accuracy and can more easily meet the requirements of surface polishing accuracy of SiC ceramics. Ultrashort-pulse lasers mainly include picosecond, femtosecond and attosecond lasers. Zhang et al. [17] used the overlapping parallel line scanning mode to achieve high-efficiency, large-area and high-precision polishing of alumina ceramics by picosecond laser and determined the ablation law and ablation threshold during the polishing process. Ihleman et al. [18] conducted polishing experiments on different oxide ceramics using nanosecond and femtosecond lasers. It was found that when using nanosecond pulsed laser polishing, the material removal mechanism was mainly plasma-induced ablation. When using femtosecond laser polishing, photochemical decomposition dominated. Since the pulse width of the femtosecond laser is extremely narrow, the interaction time of the femtosecond laser with the material is very short, and it hardly brings thermal effect to the surrounding materials. Therefore, femtosecond laser polishing is also called cold polishing. The action mechanism of cold polishing is that a single photon or multiple photons interact with the lattice or chemical bond of the material, and as a result, some components in the material are directly peeled off, that is, photochemically decomposed [19]. Kurita et al. [20] found that the number and size of deposited debris on the surface of SiC ceramics processed by femtosecond laser were much smaller than nanosecond laser processing. Taylor et al. [21] used femtosecond laser to polish SiC ceramics, by optimizing the polishing process parameters, and the problem of thermal oxidation on the surface of the material due to the high laser frequency was avoided. Chen et al. [22] reported a femtosecond laser polishing method for SiC ceramics and studied the influence of laser wavelength and pulse number on the surface morphology and composition formation mechanism. By fine-tuning the processing parameters, the subsurface defects were eliminated. After polishing, the subsurface structure was uniform and the friction coefficient was stable, and a high-quality polished surface was obtained. On the basis of single-laser polishing technology, researchers have carried out research on the composite polishing technology of laser and other energy fields. Wang et al. [23] used femtosecond laser-assisted chemical mechanical polishing of SiC crystals, and the corresponding composite polishing mechanism was explored. It was found that the surface quality and polishing efficiency of chemical mechanical polishing SiC crystals could be significantly improved when the laser process parameters were properly selected. Zheng et al. [24] proposed a new method of underwater femtosecond laser polishing for SiC ceramics, studied the influence of scanning trajectory and laser pulse energy on the surface morphology and polishing depth during underwater polishing and, finally, obtained a smooth polished surface.

In summary, researchers from different countries have carried out some investigations on laser polishing for die steel, alloy, SiC ceramics and other materials. At present, the related research on the ultrashort-pulse laser polishing mechanism and process for hard and brittle materials is still limited and needs to be further carried out. Femtosecond laser polishing has certain technical advantages for the surface precision machining of hard and brittle materials. However, there are many factors that affect the polished surface quality, among which the regulation of laser energy density has a significant impact on the polishing effect. This work studies the influence of laser energy density on femtosecond laser polishing of SiC ceramics under different working conditions in order to further promote the process improvement and technological progress of the laser polishing of hard and brittle materials. In this paper, the ablation and polishing experiments with SiC ceramics were carried out by infrared femtosecond laser, and the laser ablation threshold of SiC ceramics was calculated, and the influence of pulse energy and defocus amount on the surface morphology, surface roughness, polishing depth and oxidation degree of femtosecond laser polishing SiC ceramics were studied. The research results can guide the selection and optimization of process parameters for femtosecond laser polishing of SiC ceramics.

## 2. Materials and Methods

### 2.1. Original Materials

**Figure 1:**
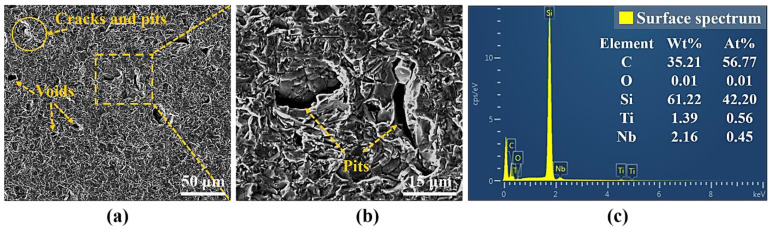
The SEM images and EDS element analysis of the initial surface. (**a**) The initial surface topography, (**b**) the partial enlarged details of the inset (**a**,**c**) the EDS elemental analysis of the inset (**b**).

The experimental samples were SiC ceramic blocks with the size of 20 mm × 20 mm × 5 mm, and which were prepared by sintering technology. The basic performance parameters of SiC ceramics are shown in Table 1. Before the experiments, the samples were put into a beaker with pure alcohol solution, and then the ultrasonic cleaning machine was used (F-020SD, FUYANG, Shenzhen, China) to clean them for 10 min. The purpose was to remove the impurities attached to the surface of the samples and avoid the impurities affecting the experimental results. The average initial surface roughness Ra = 1.1 μm of the samples was measured by the laser scanning confocal microscope (VK-X200K, KEYENCE, Osaka, Japan) at a magnification of 1000×. Then, the initial surface morphology of the experimental samples was observed by the scanning electron microscope (QUANTA FEG 250, FEI, Hillsboro, OR, USA), and the element types and contents of the initial surface were detected by EDS (FEI, Hillsboro, OR, USA), as shown in Figure 1. There was a certain porosity on the initial surface in which the content of C and Si elements were the highest.

### 2.2. Experimental Settings

**Figure 2:**
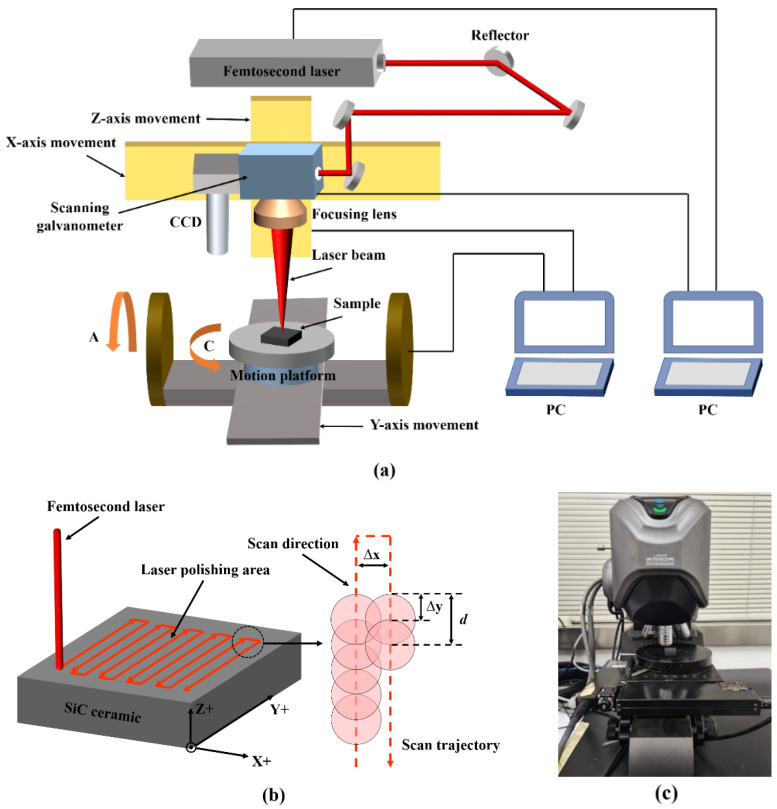
(**a**) The schematic diagram of infrared femtosecond laser processing system, (**b**) the schematic diagram of scanning trajectory and (**c**) the laser scanning confocal microscope.

In this work, experimental studies on ablation and polishing of SiC ceramics were carried out by using an infrared femtosecond laser processing system. The schematic diagrams of the infrared femtosecond laser processing system and polishing scanning trajectory are shown in Figure 2. The laser processing system is mainly composed of a femtosecond laser (HUARAY, Wuhan, China), a high-speed scanning galvanometer system, a five-axis precision motion system, an optical path system and a control system. The laser is a pulsed femtosecond laser with laser wavelength of 1064 nm, repetition frequency of 0~175 kHz, pulse width of 10~15 fs, maximum output power of 30.16 W, focusing spot diameter of 50 μm and focal length of the focusing lens of 233 mm. The cooling mode of the laser is internal circulation water cooling. In the experiments, the laser scanning confocal microscope and scanning electron microscope were used to observe the surface morphology of SiC ceramics before and after processing, the surface roughness and polishing depth were measured by VK analysis software (KEYENCE, Osaka, Japan), and elemental distribution in the machined area was detected by using EDS elemental analysis.

First, we used the laser power energy meter (OPHIR, Jerusalem, Israel) to measure the laser power, which consisted of the universal thermal power probe (50 (150) A-BB-26, OPHIR, Jerusalem, Israel) and the power meter head (VEGA, OPHIR, Jerusalem, Israel). The femtosecond laser ablation experiments were carried out on SiC ceramics under different laser powers, and the laser ablation threshold of SiC ceramics was calculated. The design of the parameters of the ablation experiments is shown in Table 2.

Then, keeping other parameters unchanged, by changing the pulse energy and defocus amount, several groups of experiments were designed. The designs of polishing experimental parameters are shown in Table 3 and Table 4. After the polishing experiments, the samples were ultrasonically cleaned with pure alcohol solution for 10 min, and then the polished surfaces were observed and detected with relevant testing instruments.

The abovementioned x-direction spot overlap ratio and y-direction spot overlap ratio can be obtained by the following formulas [25]:

(1) $$\psi_{x}=\frac{d-\Delta x}{d}$$

(2) $$\Delta y=\frac{v}{f}$$

(3) $$\psi_{y}=1-\frac{\Delta y}{d}$$

where *d* is the spot diameter (μm), Δx is the distance between adjacent scanning tracks (μm) and Δy is the distance between adjacent spots along the laser scanning direction (μm).

## 3. Results and Discussion

### 3.1. Calculation and Analysis of Femtosecond Laser Ablation Threshold of SiC Ceramics

**Figure 3:**
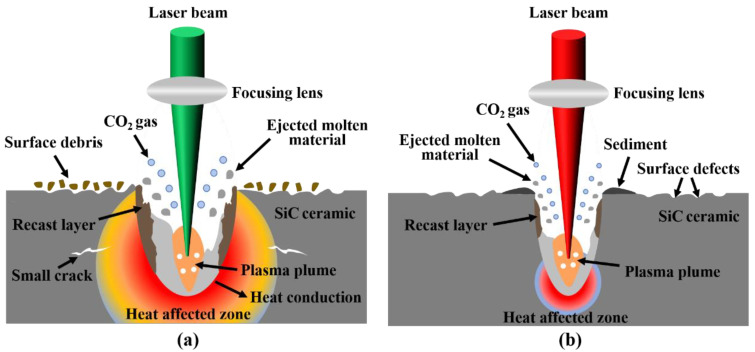
(**a**) The schematic diagram of nanosecond laser ablation of SiC ceramics and (**b**) the schematic diagram of femtosecond laser ablation of SiC ceramics.

Femtosecond laser-induced material removal is the result of the combined effect of various phenomena such as multiphoton absorption, thermal conduction, avalanche ionization, plasma expansion and liquid-phase blasting. In the case of a single pulse, the action time of the femtosecond laser is much shorter than the lattice relaxation time, and the heat energy converted by SiC ceramics after absorbing photon energy can only be conducted inside the lattice, which has almost no thermal effect on surrounding materials. In the case of multiple pulses, femtosecond laser processing also thermally affects the surrounding materials due to the thermal accumulation effect, but the thermal effect is lower than continuous wave lasers and long-pulse lasers. Figure 3 is the diagram of the action mechanism of different lasers and SiC ceramics. Observing Figure 3, it can be seen that there was recast layer on the inner wall and bottom of the ablation hole. This was because the molten material could not be discharged in time during the ablation process. After cooling and solidification, the recast layer was formed on the inner wall and bottom of the ablation hole. Related studies have shown that the recast layer formed by laser ablation of SiC ceramics is mainly composed of SiC, Si and SiO_2_, and this is because SiC ceramics undergo thermal decomposition and oxidation reactions during the laser ablation process. The relevant chemical reaction equations are as follows [26,27]:

SiC_(*l*)_ = Si_(*l*)_ + C_(*s*)_ (4)

Si_(*l*)_ + O_2(*g*)_ = SiO_2(*s*)_ (5)

C_(*s*)_ + O_2(*g*)_ = CO_2(*g*)_ (6)

The laser ablation threshold is the minimum laser energy density required to achieve material ablation removal. The calculation of the ablation threshold of SiC ceramics can guide the selection of relevant parameters in the subsequent polishing experiments, which can also avoid the phenomena of surface cracks caused by excessive laser energy or low polishing efficiency caused by too little laser energy. The equivalent diameter method was used to calculate the laser ablation threshold of SiC ceramics, which was based on the linear relationship between the square of the equivalent ablation diameter and the logarithm of the incident laser power [28,29]. Since the ablation hole was approximately circular, the width of the ablation hole was measured from the four directions of 0°, 45°, 90° and 135° along the center of the ablation hole, and then the average value of the above four width values was taken as the equivalent ablation diameter of the ablation hole.

**Figure 4:**
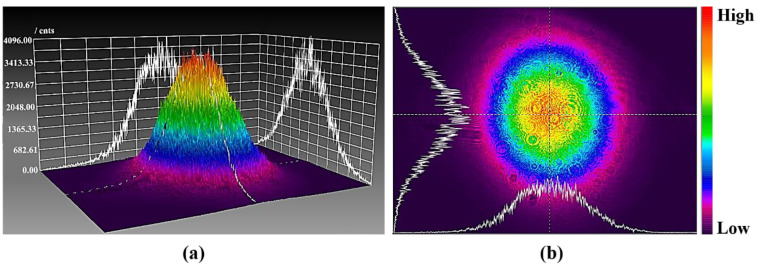
The schematic diagram of energy density distribution of femtosecond laser beam. (**a**) The 3D energy density distribution and (**b**) the 2D energy density distribution.

The infrared femtosecond laser was detected by using the beam quality analyzer (SP620U, OPHIR-SPIRICON, Jerusalem, Israel), as shown in Figure 4. The femtosecond pulsed laser beam belongs to the Gaussian beams, and its laser energy density distribution obeys Gaussian distribution, which has the characteristics of high central energy density and low surrounding energy density.

For the Gaussian beam, its energy density distribution satisfies the following relation [30]:

(7) $$\phi (r)=\phi_{0}\exp(\frac{{-2r}^{2}}{w_{0}^{2}})$$

(8) $$\phi_{0}=\frac{{2E}_{P}}{{\pi \;w}_{0}^{2}}$$

where $\phi_{0}$ represents the peak energy density of the beam center (J/cm^2^), $w_{0}$ is the beam waist radius (μm), *r* is the distance from a point in the beam to the beam center (μm) and $E_{P}$ is the laser pulse energy (μJ).

It can be seen from Equation (8) that the peak energy density of the beam center is proportional to the pulse energy. The laser pulse energy can be calculated by Equation (9):

(9) $$E_{P}=\frac{P}{f}$$

where *P* is the average laser power (W), *f* is the laser repetition frequency (kHz). Substituting Equation (9) into Equation (8), we can obtain:

(10) $$\phi_{0}=\frac{2P}{{f\;\pi \;w}_{0}^{2}}$$

Set *D* as the equivalent ablation diameter of the ablation hole (μm), $P_{\mathrm{th}}$ as the corresponding average laser power (W) when the equivalent ablation diameter is 0 μm and $\phi_{\mathrm{th}}$ as the corresponding incident laser energy density (J/cm^2^) when the equivalent ablation diameter is 0 μm, which is the laser ablation threshold. According to Equations (7) and (10), we can obtain:

(11) $$\phi_{\mathrm{th}}=\phi_{0}\exp(\frac{{-D}^{2}}{{2w}_{0}^{2}})$$

(12) $$D^{2}{=2w}_{0}^{2}(\ln\phi_{0}-\ln\phi_{\mathrm{th}})$$

(13) $$\phi_{\mathrm{th}}=\frac{{2P}_{\mathrm{th}}}{{f\;\pi \;w}_{0}^{2}}$$

Substituting Equations (10) and (13) into Equation (12), we can obtain:

(14) $$D^{2}{=2w}_{0}^{2}(\ln P+\ln\frac{2}{{\pi \;f\;w}_{0}^{2}\;\phi_{\mathrm{th}}})$$

**Figure 5:**
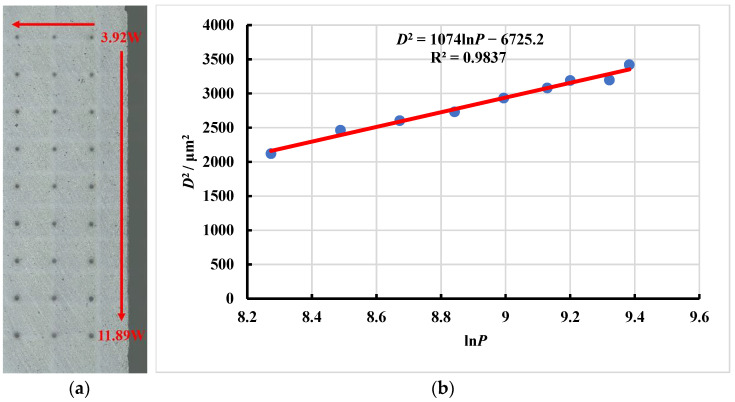
(**a**) The ablation hole distribution map and (**b**) the fitting line graph of the square of the equivalent ablation diameter and the logarithm of the incident laser power.

After the ablation experiments, the equivalent ablation diameter of the ablation hole was observed and measured by using the laser scanning confocal microscope combined with VK analysis software. The experimental data processing results are shown in Figure 5.

It can be seen from Figure 5 that the regression equation of the fitted straight line is:

(15) $$D^{2}=1074\ln P-6725.2$$

According to the above theoretical calculation formulas, it can be obtained that when the laser repetition frequency *f* = 175 kHz, wavelength *λ* = 1064 nm and ablation time *t* = 9 s, the ablation threshold of the infrared femtosecond laser ablation of SiC ceramics was 0.355 J/cm^2^.

### 3.2. The Polished Surface Quality of SiC Ceramics by Femtosecond Laser

**Figure 6:**
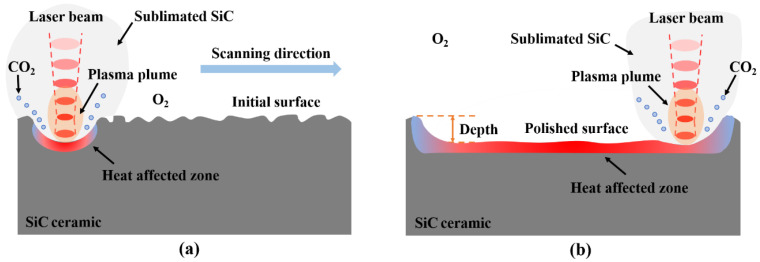
The schematic diagram of femtosecond laser polishing of SiC ceramics. (**a**) Before polishing and (**b**) after polishing.

Femtosecond laser polishing is a process in which the interaction between the laser and the material is used to remove the surface material to obtain a smoother and flatter surface. There are many factors that affect the effect of laser polishing, including the properties of the material itself, the initial surface topography, laser pulse energy, repetition frequency, scanning speed, scanning path, spot overlap rate, incident angle, defocus amount and number of scanning. In this paper, the influence of pulse energy and defocus amount on the surface characteristics of femtosecond laser polishing SiC ceramics were mainly studied. Figure 6 is the schematic diagram of the femtosecond laser polishing of SiC ceramics; due to the high energy of the femtosecond laser, the surface material is directly removed by sublimation, and the heat-affected zone is small. The laser polishing experiments were carried out in the air, and SiC ceramics chemically reacted with O_2_ in the air during polishing.

#### 3.2.1. Influence of Pulse Energy on Surface Morphology and Polishing Depth of SiC Ceramics

**Figure 7:**
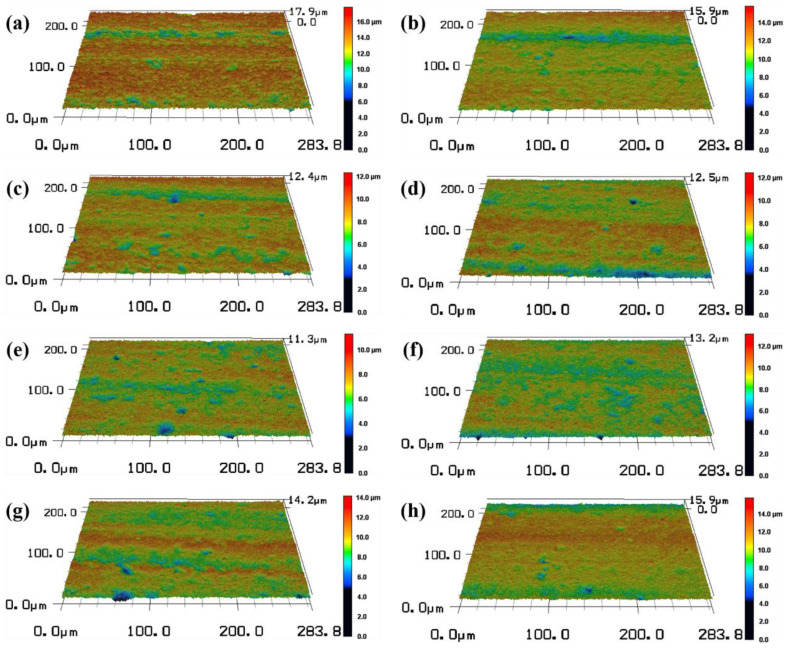
The three-dimensional morphologies of initial surface and the surface after polishing with different pulse energies (magnification 1000). (**a**) The initial surface, (**b**) 15 μJ, (**c**) 20 μJ, (**d**) 25 μJ, (**e**) 30 μJ, (**f**) 35 μJ, (**g**) 40 μJ, (**h**) 45 μJ.

Controlling the repetition frequency to remain unchanged, by changing the laser power, the laser pulse energy and laser energy density were changed. According to the femtosecond laser ablation threshold of SiC ceramics calculated previously, seven different pulse energies of 15 μJ, 20 μJ, 25 μJ, 30 μJ, 35 μJ, 40 μJ and 45 μJ were selected as the experimental variable values. Femtosecond laser polishing experiments of SiC ceramics were carried out under the conditions of laser repetition frequency of 175 kHz, scanning speed of 4812.5 mm/s, scanning spacing of 2.5 μm, defocus amount of 0 mm and the number of scanning as four times. The three-dimensional morphology of the polished surface was observed by using the laser scanning confocal microscope, as shown in Figure 7. By comparing the three-dimensional morphologies of the polished surfaces under different pulse energies, the changing trend of the surface quality could be qualitatively reflected.

**Figure 8:**
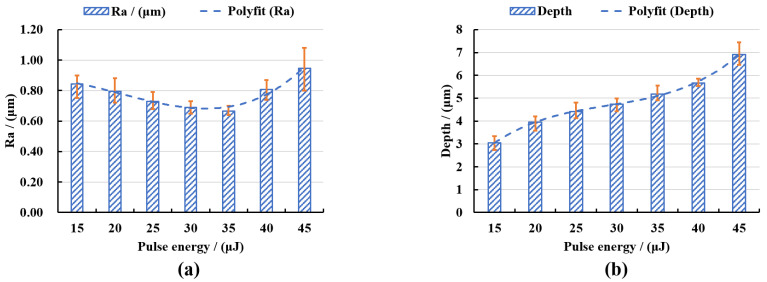
(**a**) The variation of surface roughness with pulse energy and (**b**) the variation of polishing depth with pulse energy.

In order to quantitatively analyze the surface characteristics, the surface roughness and polishing depth after different laser pulse energy polishing were measured by the laser scanning confocal microscope and VK analysis software, and the experimental data were fitted and analyzed, as shown in Figure 8. It can be seen from Figure 8a that the roughness of the polished surface first decreased with the increase of the laser pulse energy. When the pulse energy was 35 μJ, the lowest average surface roughness Ra = 0.664 μm was obtained, which was 39.64% lower than the initial average surface roughness Ra = 1.1 μm. With continued increase in the pulse energy, the surface roughness value showed an upward trend. The reasons for the above variation laws were that when the laser pulse energy was small, the corresponding laser energy density was low, which was not enough to completely remove the convex peaks on the surface of the material and so the surface was rough. When the pulse energy was increased to an appropriate value, the laser energy acted on the surface of the material uniformly, so a flatter polished surface can be obtained. When the pulse energy continued to increase, the laser energy was too large, the ablation was more serious, and the surface quality became poor. It can be seen from Figure 8b that the polishing depth showed an upward trend with the increase of the pulse energy, and when the pulse energy exceeded 40 μJ, the increased magnitude of polishing depth rose. This was because the greater the pulse energy, the greater the laser energy density, and the more energy absorbed by the material per unit area, the more intense the ablation between the laser and the material, and with greater amounts of material removed, the polishing depth thus gradually increased.

**Figure 9:**
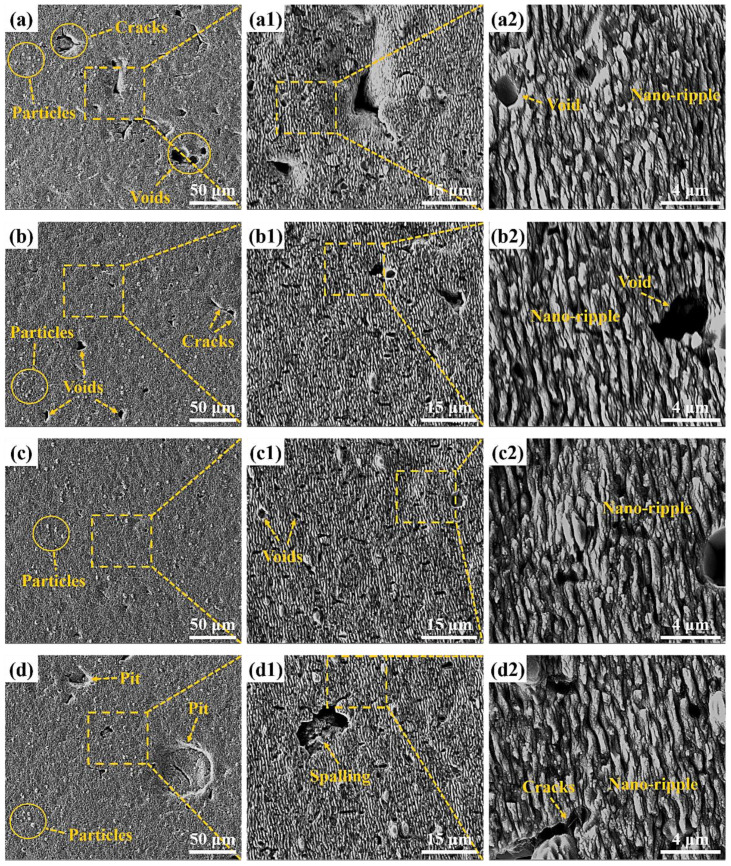
Polished surface morphologies and local magnifications under different pulse energies. (**a**) 20 μJ, (**b**) 25 μJ, (**c**) 35 μJ, (**d**) 45 μJ. The insets (**a1**), (**b1**), (**c1**) and (**d1**) were the partial enlarged details of the insets (**a**–**d**), respectively. The insets (**a2**), (**b2**), (**c2**) and (**d2**) were the partial enlarged details of the insets (**a1**), (**b1**), (**c1**) and (**d1**), respectively.

**Figure 10:**
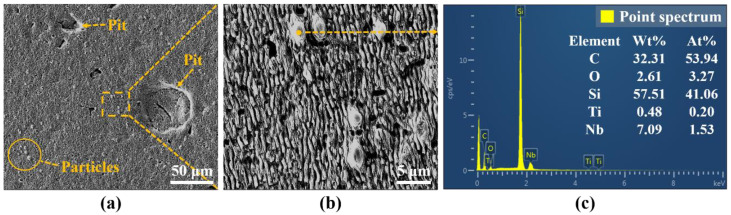
(**a**) The SEM image of polished surface corresponding to pulse energy of 45 μJ, (**b**) the partial enlarged details of the inset (**a**,**c**) the EDS elemental analysis of microparticles.

**Figure 11:**
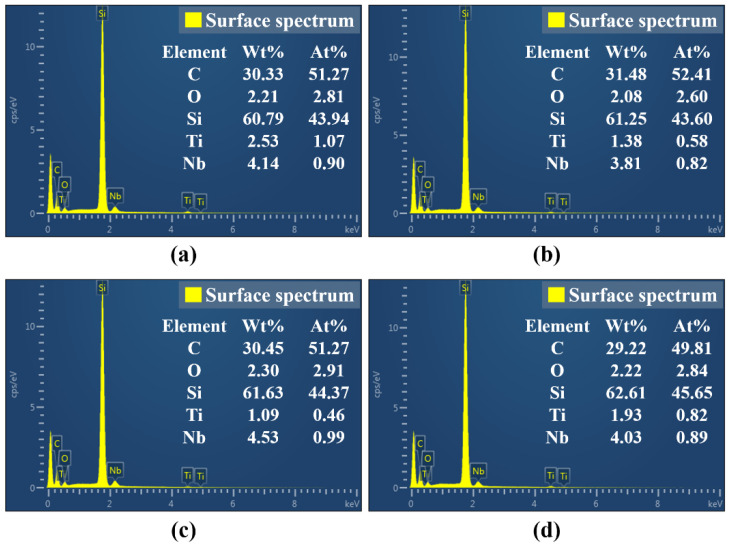
The elemental content of polished surfaces under different pulse energies. (**a**) 20 μJ, (**b**) 25 μJ, (**c**) 35 μJ, (**d**) 45 μJ. (Figures (**a**), (**b**), (**c**) and (**d**) in Figure 11 are the surface spectrum of figures (**a1**), (**b1**), (**c1**) and (**d1**) in Figure 9, respectively).

For the purpose of further study, the influence of pulse energy on the surface morphology and oxidation degree of femtosecond laser polished SiC ceramics in air environment, scanning electron microscopy was used to observe the microscopic morphology of the polished surface, and EDS was used to detect the content of elements on the polished surface, as shown in Figure 9, Figure 10 and Figure 11.

By observing Figure 1 and Figure 9, it can be found that the surface quality after polishing was improved, and the initial surface wrinkle-like morphology was basically eliminated. However, at low pulse energy, defects such as initial voids, pits and cracks were still preserved, as shown in Figure 9a, and this was because the low pulse energy was not enough to completely ablate the material. With the increase of the pulse energy, the voids on the surface became smaller and less numerous. When the pulse energy was 35 μJ, the surface quality was the best, and defects such as voids and pits were basically eliminated, as shown in Figure 9c. With continued increase in the pulse energy to 45 μJ, spalling and cracks appeared on the surface, and the surface quality deteriorated. This was because the larger pulse energy improved the ablation ability of the laser, and the interaction between the laser and the material became more intense, which led to thermal stress produced on the machined surface, resulting in brittle fracture of the material, and then the phenomena of spalling and cracks appeared. At the same time, there was a small amount of microparticles on the polished surface under the action of different pulse energies, and the periodic nano-ripple structure appeared. The periodic nano-ripple structure was induced by the laser, which was related to the laser wavelength, polarization and initial surface morphology [31,32]. The EDS point scan elemental analysis of the polished surface microparticles was performed at the pulse energy of 45 μJ, as shown in Figure 10c. Compared with the initial surface element content, it can be seen that the polished surface microparticles might be splash products during the processing, and some oxidation had occurred. Observing Figure 1c and Figure 11, it can be found that compared with the initial surface, the content of O element on the polished surface increased, indicating that there was an oxidation phenomenon in SiC ceramics polished by femtosecond laser in the air environment, and the relevant reaction equations were shown in Equations (4)–(6). Therefore, in order to eliminate the oxidation phenomenon during processing, the processing can be carried out in the environment of protective gas. It was also found that the content of each element on the polished surface under the action of different pulse energies was not much different, and the increase of pulse energy had no significant effect on the oxidation of SiC ceramics polished by femtosecond laser.

#### 3.2.2. Influence of Defocus Amount on Surface Morphology and Polishing Depth of SiC Ceramics

**Figure 12:**
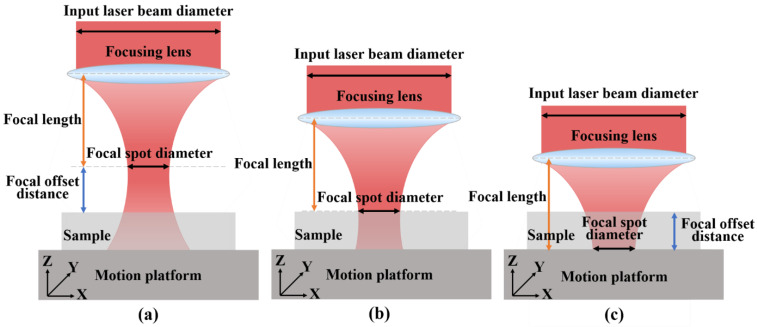
The schematic diagrams of three defocus situations of laser beam. (**a**) Positive defocus, (**b**) zero defocus and (**c**) negative defocus.

Defocus amount refers to the distance between the focal point of the laser beam and the surface to be machined of the workpiece, also known as the focus offset distance. The change in the defocus amount directly affects the size of the laser spot, which in turn affects the laser energy density in the radiation area. According to the distance between the focus of the laser beam and the surface to be machined of the workpiece, it can be divided into three cases: positive defocus, zero defocus and negative defocus. Figure 12 showed the schematic diagram of the laser beam with positive defocus, zero defocus and negative defocus.

**Figure 13:**
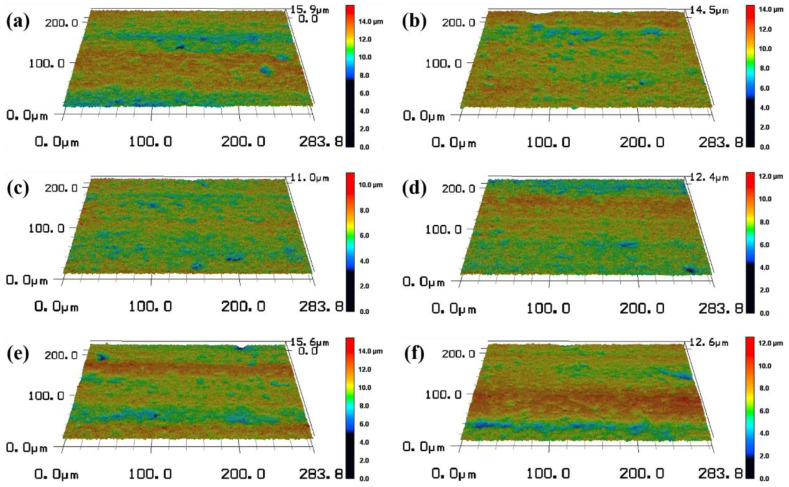
The three-dimensional topographies of the surface after polishing with different defocus amounts (magnification 1000). (**a**) −2 mm, (**b**) −1 mm, (**c**) 0 mm, (**d**) 1 mm, (**e**) 2 mm, (**f**) 3 mm.

Six different defocus amounts of −2 mm, −1 mm, 0 mm, 1 mm, 2 mm and 3 mm were selected as the experimental variable values. Femtosecond laser polishing experiments of SiC ceramics were carried out under the conditions of laser pulse energy of 30 μJ, repetition frequency of 175 kHz, scanning speed of 4812.5 mm/s, scanning spacing of 2.5 μm and the number of scanning as four times. The three-dimensional morphology of the polished surface was observed by using the laser scanning confocal microscope, as shown in Figure 13. By comparing the three-dimensional morphologies of the polished surfaces under different defocus amounts, the changing trend of the surface quality could be qualitatively reflected.

**Figure 14:**
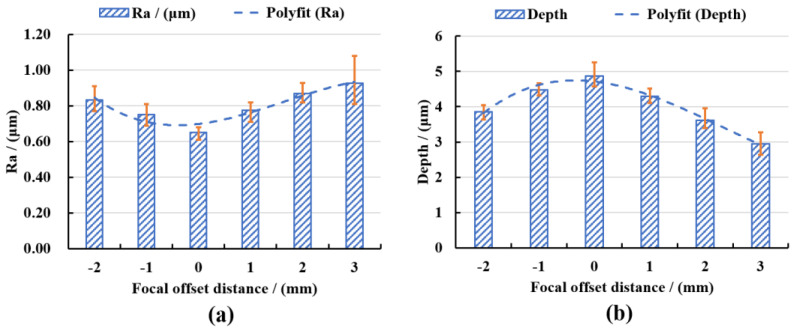
(**a**) The variation of surface roughness with defocus amount and (**b**) the variation of polishing depth with defocus amount.

In order to quantitatively analyze the surface characteristics, the surface roughness and polishing depth after different defocus amounts polishing were measured by the laser scanning confocal microscope and VK analysis software, and the experimental data were fitted and analyzed, as shown in Figure 14.

It can be seen from Figure 14a that the polished surface roughness increased with the increase in the defocus amount whether it was positive defocus or negative defocus, and in the case of positive defocus, with the increase in the defocus amount, the increase in the surface roughness gradually slowed down. When the defocus amount was 0 mm, the lowest average surface roughness Ra = 0.652 μm was obtained, which was 40.73% lower than the initial average surface roughness Ra = 1.1 μm. The increase in the defocus amount leads to a larger laser spot irradiated on the workpiece surface, a decrease in the laser energy density and a weakening of the material removal ability. It is well-known that when other parameters remain unchanged, and the laser energy density is inversely proportional to the diameter of the laser spot, so the energy density in the laser spot irradiated on the workpiece surface gradually decreases with the increase in the defocus amount. The lower laser energy density is not enough to completely remove the convex peaks on the surface of the material, and the surface roughness is larger. It can be seen from Figure 14b that when the defocus amount was 0 mm, the maximum average polishing depth of 4.874 μm was obtained. In the positive defocus state, the polishing depth decreased with the increase in the defocus amount. This was because the larger defocus amount, the larger diameter of the laser spot, resulting in smaller laser energy density resulting in less laser energy absorbed by the material per unit area, which resulted in continuous weakening of the laser ablation processing capability, so the polishing depth gradually decreased. It can also be found in Figure 14 that when the defocus amounts were −1 mm and 1 mm, both the average roughness value of the polished surface and polishing depth were basically the same. This was because positive and negative defocus of equal magnitude had similar laser energy density and processing capacity, so the polishing results were similar for both.

**Figure 15:**
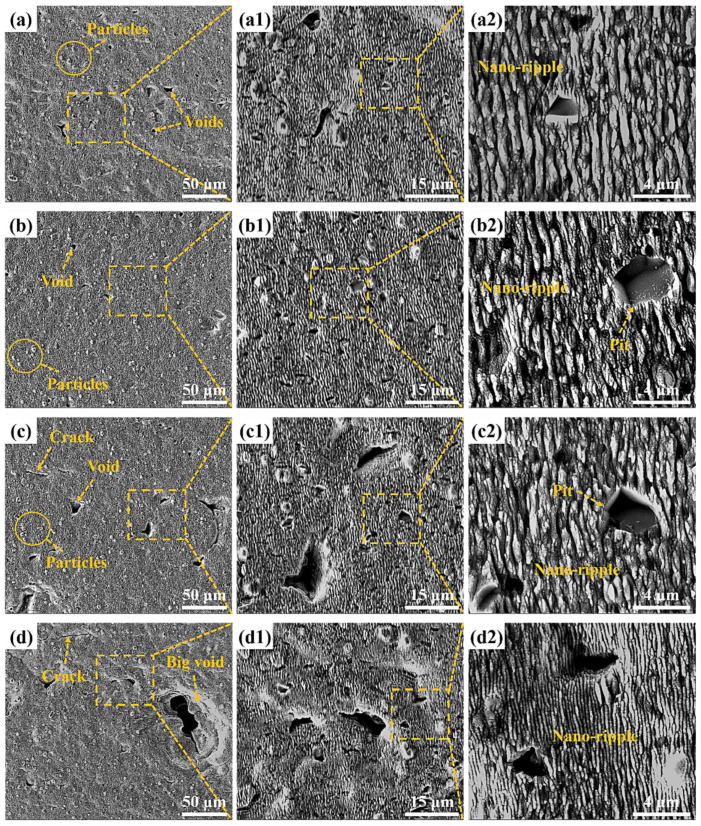
Polished surface morphologies and local magnifications under different defocus amounts. (**a**) −1 mm, (**b**) 0 mm, (**c**) 2 mm, (**d**) 3 mm. The insets (**a1**), (**b1**), (**c1**) and (**d1**) were the partial enlarged details of the insets (**a**), (**b**), (**c**) and (**d**), respectively. The insets (**a2**), (**b2**), (**c2**) and (**d2**) were the partial enlarged details of the insets (**a1**), (**b1**), (**c1**) and (**d1**), respectively.

**Figure 16:**
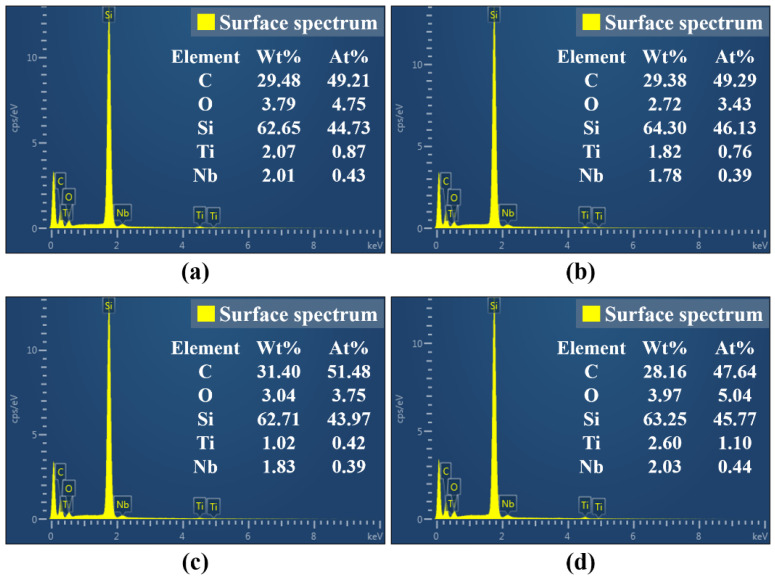
The elemental content of polished surfaces under different defocus amounts. (**a**) −1 mm, (**b**) 0 mm, (**c**) 2 mm, (**d**) 3 mm. (Figures (**a**), (**b**), (**c**) and (**d**) in Figure 16 are the surface spectrum of figures (**a1**), (**b1**), (**c1**) and (**d1**) in Figure 15, respectively).

In order to further study the influence of defocus amount on the surface morphology and oxidation degree of femtosecond laser polished SiC ceramics in an air environment, scanning electron microscopy was used to observe the microscopic morphology of the polished surface, and EDS was used to detect the content of elements on the polished surface, as shown in Figure 15 and Figure 16.

It can be seen from Figure 15 that the surface defects were the least and the surface quality was the best under the zero defocus condition. Compared with the initial surface, the polished surface roughness of the abovementioned positive and negative defocus states was reduced. However, the polished surface retained the defects of the initial surface, e.g., voids, cracks and pits. With the increase of the absolute value of defocus amount, the surface defects became more obvious and the surface quality was poor. The larger the defocus amount is, the larger the spot diameter is. The smaller the laser energy density is, the more the removal of the surface material is weakened by the low energy density. At the same time, microparticles and periodic nano-ripple structures were generated on the polished surface. When the defocus amount was 3 mm, the periodic nano-ripples generated on the polished surface were significantly finer, as shown in Figure 15(d2). Observing Figure 1c and Figure 16, it can be found that oxidation occurred during the polishing process, and the element contents of the polished surfaces with different defocus amounts were not much different. However, the content of the O element on the polished surface in the defocus states was slightly higher than that in the focus state.

## 4. Conclusions

In this paper, in order to further promote the process improvement and technological progress of femtosecond laser polishing of SiC ceramics, the interaction mechanism between femtosecond laser and SiC ceramics and the influence of laser energy density on femtosecond laser polishing of SiC ceramics under different working conditions were studied. The infrared femtosecond laser was used to ablate and polish SiC ceramics. Then, the equivalent diameter method was used to calculate the laser ablation threshold of SiC ceramics, and the influence of pulse energy and defocus amount on the surface morphology, surface roughness, polishing depth and oxidation degree of femtosecond laser polishing SiC ceramics were investigated. The main conclusions are as follows:

(1) Based on the linear relationship between the square of the equivalent ablation diameter and the logarithm of the incident laser power, the ablation threshold of SiC ceramics was deduced and calculated by the equivalent diameter method. It was obtained that when the laser repetition frequency *f* = 175 kHz, wavelength *λ* = 1064 nm and ablation time *t* = 9 s, the laser ablation threshold of SiC ceramic is 0.355 J/cm^2^. The beam quality analyzer was used to verify that the laser energy density distribution of the femtosecond laser beam obeyed the Gaussian distribution, with the characteristics of high central energy density and low peripheral energy density.
(2) Compared with the initial surface, the surface quality of polished surface was improved. In the case of low pulse energy, the laser energy was not enough to completely remove the surface material, and the polished surface still retained initial surface defects such as voids, pits and cracks, so the surface roughness was high. With the increase in pulse energy, the surface roughness decreased first. When the pulse energy was 35 μJ, the polished surface quality was the best, and the lowest average surface roughness, Ra = 0.664 μm, was obtained. With continued increase in the pulse energy, spalling and cracks appeared on the polished surface, the surface quality deteriorated, and the surface roughness value showed an upward trend. The phenomena of spalling and cracks were caused by the intense ablation of the laser and the material under the high pulse energy, and the thermal stress on the surface caused the brittle fracture of the material. The greater the pulse energy, the more energy absorbed by the material per unit area and the more the material removal, the overall increase in the trend of the polishing depth correlated with the increase in the pulse energy. There were periodic nano-ripple structures that appeared on the polished surface, and oxidation phenomenon occurred. The change of pulse energy had no significant effect on the oxidation phenomenon of the SiC ceramics polished by femtosecond laser.
(3) Under the condition of constant laser pulse energy, repetition frequency, scanning speed, scanning spacing and the number of scanning, the surface roughness increased with the increase in the absolute value of the defocus amount, and the polishing depth decreased with the increase in the absolute value of the defocus amount. With the increase in the absolute value of the defocus amount, the laser spot irradiated on the surface of the workpiece became larger, which would further reduce the laser energy density. The low laser energy density could not completely remove the surface material, the material removal amount was small, and the initial surface defects were retained, so the surface quality gradually deteriorated, and the polishing depth gradually decreased. In the case of zero defocus, the lowest average surface roughness, Ra = 0.652 μm, and the maximum average polishing depth of 4.874 μm were obtained. At the same time, the periodic nano-ripple structures were generated on the polished surface, and when the defocus amount was 3 mm, the size of the periodic nano-ripple structures generated on the polished surface was significantly smaller. A small amount of oxidation also occurred during the polishing process with variable defocus amounts, and the change of defocus amount had no significant effect on the oxidation phenomenon of the SiC ceramics polished by femtosecond laser polishing.

## Figures and Tables

**Table 1:** The performance parameters of SiC ceramic materials.

Density	Flexural Strength	Elastic Modulus	Thermal Expansion Coefficient	Thermal Conductivity	Microhardness
3200 kg/m^3^	500 MPa	420 GPa	4.2 (1 × 10^−6^/K)	60 W/(m·K)	2500 HV

**Table 2:** The femtosecond laser ablation experimental parameters of SiC ceramics.

Number	Laser Frequency *f*/kHz	Ablation Time *t*/s	Laser Power *P*/mW
1	175	9	3920
2	175	9	4860
3	175	9	5840
4	175	9	6920
5	175	9	8060
6	175	9	9220
7	175	9	9900
8	175	9	11,180
9	175	9	11,890

**Table 3:** Experimental parameters of femtosecond laser polishing of SiC ceramics with variable pulse energy.

Parameter	Value						
Laser frequency, *f*/kHz	175						
Defocus amount, *h*/mm	0						
Scanning spacing, Δx/μm	2.5						
Spot overlap ratio in the x-direction, *ψ*_x_/(%)	95						
Scanning speed, *v*/(mm/s)	4812.5						
Spot overlap ratio in the y-direction, *ψ*_y_/(%)	45						
Number of scanning, n	4						
Laser power, *P*/W	2.625	3.5	4.375	5.25	6.125	7	7.875
$\mathrm{Pulse}\;\mathrm{energy},\;E_{P}$/μJ	15	20	25	30	35	40	45

**Table 4:** Experimental parameters of femtosecond laser polishing of SiC ceramics with variable defocus amount.

Parameter	Value					
Laser frequency, *f*/kHz	175					
Laser power, *P*/W	5.25					
$\mathrm{Pulse}\;\mathrm{energy},\;E_{P}$/μJ	30					
Scanning spacing, Δx/μm	2.5					
Spot overlap ratio in the x-direction, *ψ*_x_/(%)	95					
Scanning speed, *v*/(mm/s)	4812.5					
Spot overlap ratio in the y-direction, *ψ*_y_/(%)	45					
Number of scanning, n	4					
Defocus amount, *h*/mm	−2	−1	0	1	2	3

## Data Availability

Not applicable.
